# Mischief Makers: Secondhand Smoke, Lead Linked to Conduct Disorder

**DOI:** 10.1289/ehp.116-a307b

**Published:** 2008-07

**Authors:** Cynthia Washam

A study of 3,081 U.S. children now confirms the results of smaller studies associating exposure to secondhand tobacco smoke and lead with conduct disorder (CD), a persistent disruptive behavior pattern that includes aggression, lying, stealing, and destruction of property **[*EHP* 116:956–962; Braun et al.]**. According to the study authors, postnatal lead exposure and both pre- and postnatal exposure to secondhand smoke can significantly increase the risk of CD in children. Children with CD face a higher risk of anxiety disorders and substance abuse.

Using data from the National Health and Nutrition Examination Survey (NHANES) 2001–2004, the researchers obtained a nationally representative sample of children aged 8–15 years (earlier studies by other researchers relied on smaller regional samples collected from clinic visits). Caregiver responses to a questionnaire known as the Diagnostic Interview Schedule for Children determined whether children met CD diagnositic criteria established by the *Diagnostic and Statistical Manual of Mental Disorders IV*. Prenatal exposure to secondhand smoke was estimated based on caregiver responses to questions regarding smoking habits during pregnancy; postnatal exposure was measured using blood serum concentrations of cotinine, a nicotine metabolite. Lead exposure was determined by measuring blood lead concentrations.

The research team identified CD in 2.1% of the subjects, corroborating earlier prevalence estimates. The team determined that children exposed prenatally to secondhand smoke were 3-fold more likely to meet CD diagnostic criteria than those not exposed. Youngsters exposed postnatally to the highest levels of secondhand smoke were 9.15 times more likely to exhibit CD symptoms than those exposed to the lowest levels.

The research team also examined the association between behavior and lead exposure, the results of which support those of previous studies suggesting that elevated blood lead also is a risk factor for CD. Children with blood lead levels higher than 1.5 μg/dL were 8.64 times more likely to have met CD diagnostic criteria in the past year than children with levels lower than 0.7 μg/dL.

Limitations to the study include inability to infer causal relationships because of the cross-sectional nature of the data. In addition, the reported prevalence of CD could be underestimated because caregivers completing the DISC module may not recognize disruptive behaviors in their children. Nevertheless, the authors suggest that millions of children are currently being exposed to levels of secondhand smoke and lead that could increase the risk for persistent, disruptive, and even violent behavior, despite reductions in recent years in children’s exposure to these toxicants.

## Figures and Tables

**Figure:**
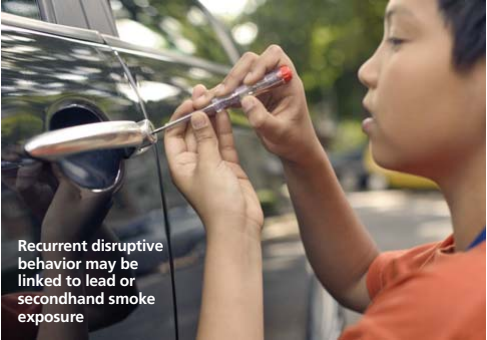
Recurrent disruptive behavior may be linked to lead or secondhand smoke exposure

